# Genetics Evaluation of Targeted Exome Sequencing in 223 Chinese Probands With Genetic Skeletal Dysplasias

**DOI:** 10.3389/fcell.2021.715042

**Published:** 2021-09-07

**Authors:** Shanshan Lv, Jiao Zhao, Lei Xi, Xiaoyun Lin, Chun Wang, Hua Yue, Jiemei Gu, Weiwei Hu, Wenzhen Fu, Zhanying Wei, Hao Zhang, Yunqiu Hu, Shanshan Li, Zhenlin Zhang

**Affiliations:** Shanghai Clinical Research Center of Bone Disease, Department of Osteoporosis and Bone Disease, Shanghai Jiao Tong University Affiliated Sixth People’s Hospital, Shanghai, China

**Keywords:** targeted exome sequencing, genetic skeletal dysplasia, molecular diagnosis, genetics evaluation, clinical utility

## Abstract

Genetic skeletal dysplasias (GSDs) are a type of disease with complex phenotype and high heterogeneity, characterized by cartilage and bone growth abnormalities. The variable phenotypes of GSD make clinical diagnosis difficult. To explore the clinical utility of targeted exome sequencing (TES) in the diagnosis of GSD, 223 probands with suspected GSD were enrolled for TES with a panel of 322 known disease-causing genes. After bioinformatics analysis, all candidate variants were prioritized by pathogenicity. Sanger sequencing was used to verify candidate variants in the probands and parents and to trace the source of variants in family members. We identified the molecular diagnoses for 110/223 probands from 24 skeletal disorder groups and confirmed 129 pathogenic/likely pathogenic variants in 48 genes. The overall diagnostic rate was 49%. The molecular diagnostic results modified the diagnosis in 25% of the probands, among which mucopolysaccharidosis and spondylo-epi-metaphyseal dysplasias were more likely to be misdiagnosed. The clinical management of 33% of the probands also improved; 21 families received genetic counseling; 4 families accepted prenatal genetic diagnosis, 1 of which was detected to carry pathogenic variants. The results showed that TES achieved a high diagnostic rate for GSD, helping clinicians confirm patients’ molecular diagnoses, formulate treatment directions, and carry out genetic counseling. TES could be an economical diagnostic method for patients with GSD.

## Introduction

Genetic skeletal dysplasia (GSD) is a diverse group of bone and cartilage disorders that are manifested as abnormal growth, development, and morphometry; this condition has diverse clinical presentations and high genetic heterogeneity (Krakow and Rimoin, 2010). The clinical manifestations range from slight skeletal changes to severe bone deformity, even threatening patients’ lives in some cases. Many forms of skeletal dysplasia result in short stature (proportionate or disproportionate) and skeletal abnormalities and involve multiple organ systems, such as the nervous, visual, and auditory systems. Although each type of skeletal dysplasia is relatively rare, the total quantity is considerable. Early statistics show that skeletal dysplasia has a collective birth incidence of almost 1/5,000 in United States (Orioli et al., 1986). Although no population-based studies have been conducted in China to determine the prevalence of skeletal dysplasia, there is no doubt at present that China accounts for a large share of rare-disease cases in the world (Wang et al., 2010).

Currently, the diagnosis of GSD is based on clinical, radiological, biochemical, and molecular criteria. However, most patients have not received adequate diagnosis and therapy due to clinicians’ limited experience in diagnosis of GSD. Especially in China, there are no official data on the definition of skeletal dysplasia, and there is little information in relevant epidemiological records. Therefore, Chinese clinicians are not particularly conversant with those diseases. China’s definite diagnosis rate is relatively low compared with those of other countries. One study revealed that only 5% of the reported osteogenesis imperfecta (OI) cases in the China Biomedical Database (CBM) had been identified by exact type (Cui et al., 2012). Making a definite diagnosis of GSD has become a major task for us at present.

The 9th edition of the nosology and classification of genetic skeletal diseases contains 436 different diseases and 42 groups, and the number of causative genes has increased to 364 since the previous edition (Bonafe et al., 2015). To date, approximately 92% of GSD cases have been described along with their causative variants, which is attributable to the continuous innovation of next-generation sequencing (NGS) technology. NGS enables quick sequencing of a large number of candidate genes at one time; it is noticeably less time consuming than Sanger sequencing (Sobreira et al., 2010). The technical simplicity of NGS allows it to be used on a large scale in the study and diagnosis of monogenic diseases (Bamshad et al., 2011; Yang et al., 2013) through testing methods including targeted exome sequencing (TES), whole exome sequencing (WES), and whole genome sequencing (WGS). WES and WGS can conduct a comprehensive exploration of genes, which is significant for researchers exploring unknown disease-causing genes (Min et al., 2011; Cameron-Christie et al., 2018). However, WES and WGS are costly, and the large amounts of resulting data are difficult for professionals to analyze. Managing and storing those data is also a challenge. In contrast, TES has the advantages of short turnaround time, a relatively low price, and deeper coverage. As TES only focuses on the targeted exons, in the same total reads, it could achieve deeper coverage and improve the sensitivity and specificity of the analysis (Mamanova et al., 2010). Therefore, we used TES as our first choice to detect GSD.

The purpose of this study was to evaluate the clinical utility of TES (containing 322 known causative gene) in 223 probands with suspected GSD. We assessed the diagnostic rate of TES, analyzed the modification of diagnoses after TES, and summarized the impact of molecular diagnosis on probands. Our results demonstrated that TES is an economical method for GSD diagnosis.

## Materials and Methods

### Subjects

This study was approved by the Ethics Committee of Shanghai Jiao Tong University Affiliated Sixth People’s Hospital (SP-2019-117). All recruited probands or their legal guardians provided written informed consent. Probands were selected from the database of Shanghai Clinical Research Center of Bone Diseases, which was established by the Department of Osteoporosis and Bone Diseases at Shanghai Jiao Tong University Affiliated Sixth People’s Hospital in 2010. The inclusion criteria were as follows: (1) proportionate or disproportionate short stature (asymmetric shortening of trunk or limb length); (2) unexplained bone pain, skeletal deformity, fragility fractures, or abnormal bone density, especially with other system abnormalities (hearing loss, abnormal teeth, etc.); (3) x-rays showing abnormal vertebral body shape, irregular epiphyses, rough and calcified metaphyses, and abnormally long-bone diaphyses; (4) laboratory tests showing abnormal indicators related to bone metabolism; (5) an early age of onset (childhood or after birth), a family history, or closely consanguineous parents. Probands needed to meet more than one criterion to be enrolled. Some recognizable causes of skeletal dysplasia had been excluded through preliminary examinations, for example, long-term use of drugs that affect bone metabolism (such as glucocorticoids, adrenaline, anabolic steroid hormones, or anticonvulsants), bone manifestations caused by disorders of other systems (such as nephrotic syndrome, chronic renal failure, renal tubule acidosis, Fanconi syndrome, or hyperparathyroidism), and bone dysplasia caused by nutritional deficiency (such as insufficient vitamin D intake or disorders of absorption and metabolism).

**FIGURE 1:**
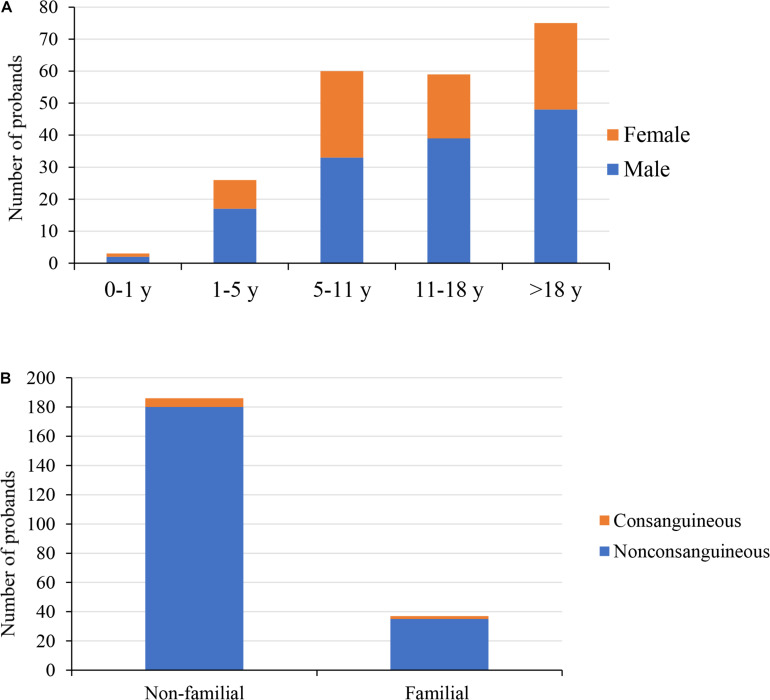
Demographics of the 223 probands. **(A)** The distribution of gender and age of 223 probands; male accounted for 62%. **(B)** Family history and parental consanguinity of 223 probands; 4% probands were born to consanguineous parents. y, years.

Ultimately, we enrolled 223 probands, collected detailed medical history data (including previous visit information and family history), and performed improved blood biochemical examinations and imaging. Based on clinical, biological, and imaging results, clinicians gave a preliminary clinical diagnosis. As some probands had undergone Sanger sequencing prior to the present study, we divided probands into three categories based on genetic testing, as follows: Probands series 1: probands had not undergone genetic sequencing before; Probands series 2: probands had undergone Sanger sequencing at least once, but no pathogenic variant was found; Probands series 3: probands had clearly pathogenic variant(s) confirmed previously by Sanger sequencing. This group contains 44 verified variants for evaluation of the sensitivity and specificity of TES. The demographics, phenotype descriptions, and genetic tests are summarized in Figure 1 and Supplementary Table 1.

### Targeted Exome Sequencing and Variant Analysis

Peripheral blood samples were collected from probands and their available family members. We used a QuickGene DNA whole blood kit (Kurabo Industries Ltd., Osaka, Japan) and a Nucleic Acid Isolation system (QuickGene-610L; AutoGen, Inc., Holliston, MA, United States) to extract genomic DNA. We designed a gene capture array (SureSelect Reagent kit; Agilent Technologies, Santa Clara, CA, United States) containing 322 genes (Supplementary Table 2), which is based on the 2015 revision of the nosology and classification of genetic skeletal disorders (Bonafe et al., 2015). A DNA library was constructed, and DNA fragments were sorted and purified. High-throughput sequencing was performed with an Illumina HiSeq-NovaSeq (Illumina, San Diego, CA, United States) to generate FastQ files. BWA (Li and Durbin, 2009) and Picard software were used for reference sequence alignment analysis, and samples with poor sequencing quality were excluded. The average sequencing depth of the original data of each sample was above 300×, and the base Q30 ratio was 91%. Sequencing quality information is provided in Supplementary Table 3.

The GATK HaplotypeCaller method was used to detect the SNVs and indels of each sample, and the variants were prioritized and filtered by the software according to the defined criteria. The allele frequency of SNVs and indels were evaluated by comparison with variant databases (including 1000 Genomes, ESP6500, and gnomAD). The conservation of SNVs and indels and their deleterious effects on the corresponding proteins were predicted by *in silico* tools (including MutationTaster, PolyPhen-2, SIFT, and CADD). We preferentially selected variants that met the following conditions: (1) non-synonymous variants located in exons or splicing regions; (2) SNVs whose allele frequency was lower than 0.001; (3) highly conservative SNVs that were predicted to be pathogenic; (4) known pathogenic variants in HGMD. These selected variants were associated with clinical phenotypes, imaging findings and genetic patterns to identify candidate variants. Sanger sequencing was used for validation and was also performed in family members to find the source of variation. Candidate variants were classified by following the guidelines of the American College of Medical Genetics and Genomics and the Association for Molecular Pathology (ACMG/AMP) (Richards et al., 2015). We defined “diagnostic yield” as the proportion of probands who received a molecular diagnosis.

## Results

### Description of the Cohort

In this study, 223 probands with suspected GSD underwent the TES. This cohort was predominantly male (139/223, 62%). Most of the probands were children and young adults (Figure 1A); the median age at referral for testing was 13 years (age range: 4 months to 59 years old), and the average age of onset was 5 years. In this cohort, 8/223 (4%) probands had consanguineous parents; 37/223 (17%) probands had a family history (Figure 1B), with a total of 40 affected family members. We were unable to obtain peripheral blood from the parents of 21 probands, including seven affected family members. One family was unavailable due to divorce, three were deceased, and the rest refused to provide peripheral blood. In the present study, the most common initial clinical diagnosis was OI (70/223, 31%), followed by spondyloepiphyseal dysplasia (36/223, 16%) and hypophosphatemic rickets (24/223, 11%, Table 1).

**TABLE 1:** Clinical diagnoses of 223 probands who were suspected with genetic skeletal dysplasia.

Clinical diagnoses of probands	*n*	(%)
Osteogenesis imperfecta	78	35%
Spondyloepiphyseal dysplasia	35	16%
Hypophosphatemic rickets	23	10%
Hypertrophic osteoarthropathy	12	5%
Mucolipidosis	10	5%
Brachydactyly, polydactyly	9	4%
Osteosclerosis	8	4%
Spondylometaphyseal dysplasia	7	3%
Epiphyseal dysplasia	5	2%
Metaphyseal dysplasia	5	2%
Cleidocranial dysplasia	4	2%
Progressive pseudorheumatoid dysplasia	3	1%
Bone diseases with atypical clinical phenotypes	24	11%

### Characteristics of the Variant Spectrum

**FIGURE 2:**
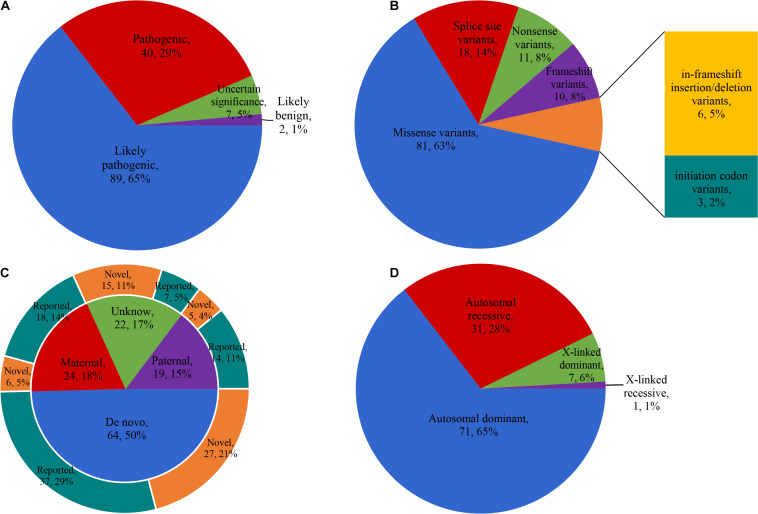
Characteristics of the variants detected. **(A)** The classification of 138 variants according to ACMG/AMP guidelines. **(B)** Mutation types of 129 pathogenic/likely pathogenic variants. **(C)** Genetic origin of 129 pathogenic/likely pathogenic variants and the proportion of reported and novel. **(D)** The disease inheritance pattern of 110 probands with clear molecular diagnosis. ACMG/AMP, American College of Medical Genetics and Genomics and the Association for Molecular Pathology.

After preliminary filtration, we obtained 138 variants in 48 candidate genes. Sanger sequencing was performed in 114 families. All 138 variants were confirmed by Sanger sequencing, which excluded false positives. According to ACMG/AMP guidelines, 129 variants were classified as pathogenic/likely pathogenic, seven variants were classified as having uncertain significance, and two variants were classified as benign/likely benign (Figure 2A). Among 129 pathogenic/likely pathogenic variants, 81 were missense variants, 18 were splice-site variants, 11 were nonsense variants, 10 were frameshift variants, six were in-frameshift insertion/deletion variants, and three were initiation codon variants (Figure 2B). The test results showed that 64 (50%) variants were *de novo*, 24 (19%) were paternal, 19 (15%) were maternal, and 22 (17%) were of unknown origin (Figure 2C). After reviewing the literature and combining the report of the Human Gene Mutation Database in 2020, 75 variants were reported, and 54 variants were novel.

### Diagnostic Yield

We clarified the molecular diagnosis of 110 probands and confirmed 48 genes (cause/likely cause GSD) from 24 skeletal disorder groups (Table 2). The disease inheritance patterns of these 110 probands were autosomal dominant (*n* = 71), autosomal recessive (*n* = 31), X-linked dominant (*n* = 7), and X-linked recessive (*n* = 1, Figure 2D). The total diagnostic rate was 49%.

**TABLE 2:** Molecular diagnostic classification of 110 probands based on the 2015 revision of the nosology and classification of genetic skeletal disorders.

ID	Group of disorders	Name of disorder	Inheritance	Gene	Number
1	FGFR3 chondrodysplasia group	Hypochondroplasia	AD	FGFR3	6
2	Type 2 collagen group	Spondyloepiphyseal dysplasia	AD	COL2A1	8
		Spondyloepiphyseal dysplasia congenita	AD	COL2A1	3
		Hypochondrogenesis	AD	COL2A1	1
		Stickler syndrome	AD	COL2A1	1
		Kniest dysplasia	AD	COL2A1	1
4	Sulfation disorders group	Achondrogenesis type 1B	AR	SLC26A2	1
5	Perlecan group	Schwartz-Jampel syndrome, type 1	AR	HSPG2	1
6	Aggrecan group	Spondyloepiphyseal dysplasia, Kimberley type	AD	ACAN	2
7	Filamin group and related disorders	Larsen syndrome	AD	FLNB	2
8	TRPV4 group	Spondyloepimetaphyseal dysplasia, Maroteaux type	AD	TRPV4	2
		Spondylometaphyseal dysplasia, Kozlowski type	AD	TRPV4	1
9	Ciliopathies with major skeletal involvement	Ellis-van Creveld syndrome	AR	EVC2	1
10	Multiple epiphyseal dysplasia and pseudoachondroplasia group	Multiple epiphyseal dysplasia	AD	COL9A3	1
		Multiple epiphyseal dysplasia	AD	COMP	1
		Pseudoachondroplasia	AD	COMP	3
11	Metaphyseal dysplasias	Metaphyseal chondrodysplasia, Schmid type	AD	COL10A1	2
		Metaphyseal anadysplasia type 1	AD	MMP13	1
20	Dysplasias with multiple joint dislocations	Spondyloepimetaphyseal dysplasia with joint laxity	AD	KIF22	1
21	Chondrodysplasia punctata (CDP) group	CDP, X-linked recessive, brachytelephalangic type	XLR	ARSE	1
23	Osteopetrosis and related disorders	Osteopetrosis, autosomal dominant type 2	AD	CLCN7	3
		Pycnodysostosis	AR	CTSK	1
		Osteopetrosis, autosomal recessive	AR	TCIRG1	1
24	Other sclerosing bone disorders	Osteopoikilosis	AD	LEMD3	1
		Osteopetrosis, autosomal dominant I	AD	LRP5	1
		Hypertrophic osteoarthropathy	AR	SLCO2A1	3
		Camurati-Engelmann disease	AD	TGFB1	2
25	Osteogenesis imperfecta and decreased bone density group	Osteogenesis imperfecta I	AD	COL1A1	6
		Osteogenesis imperfecta I	AD	COL1A2	5
		Osteogenesis imperfecta IV	AD	COL1A2	3
		Osteogenesis imperfecta V	AD	IFITM5	3
		Osteogenesis imperfecta VII	AR	CRTAP	1
		Osteogenesis imperfecta XI	AR	FKBP10	1
		Osteogenesis imperfecta XIII	AR	TMEM38B	1
		Osteogenesis imperfecta XIV	AR	BMP1	2
		Osteoporosis-pseudoglioma syndrome	AR	LRP5	1
		Cole-Carpenter syndrome 1	AD	P4HB	1
		Osteoporosis, X-linked	XLD	PLS3	2
		Cole-Carpenter syndrome 2	AR	SEC24D	2
26	Abnormal mineralization group	Hypophosphatemic rickets, X-linked	XLD	PHEX	4
		Hypophosphatemic rickets, autosomal recessive, type 1	AR	DMP1	2
		Hypophosphatasia	AR	ALPL	1
		Hypophosphatemic rickets with hypercalciuria	AR	SLC34A3	1
		Dent disease	XLD	CLCN5	1
27	Lysosomal storage diseases with skeletal involvement (dysostosis multiplex group)	Mucopolysaccharidosis IVa	AR	GALNS	6
		Mucolipidosis III	AR	GNPTAB	2
		Mannosidosis, alpha	AR	MAN2B1	1
28	Osteolysis group	Hajdu-Cheney syndrome	AD	NOTCH2	1
29	Disorganized development of skeletal components group	Fibrodysplasia ossificans progressiva	AD	ACVR1	1
30	Overgrowth (tall stature) syndromes with skeletal involvement	Loeys-Dietz syndrome 4	AD	TGFB2	1
32	Cleidocranial dysplasia and related disorders	Cleidocranial dysplasia	AD	RUNX2	2
31	Genetic inflammatory/rheumatoid-like osteoarthropathies	Progressive pseudorheumatoid dysplasia	AD	WISP3	2
37	Brachydactylies (without extraskeletal manifestations)	Brachydactyly type A2	AD	GDF5	1
		Brachydactyly type D	AD	HOXD13	1
41	Polydactyly-syndactyly-triphalangism group	Preaxial polydactyly type 4	AD	GLI3	1
	Not classified	Short stature with non-specific skeletal abnormalities	AD	NPR2	1
	Not classified	Hereditary motor neuropathy	AD	TRPV4	1

*AD, autosomal dominant inheritance; AR, autosomal recessive inheritance; XLD, X-linked dominant inheritance; XLR, X-linked recessive inheritance.*

Because some probands had previously received genetic testing, we divided the enrolled probands into three groups based on the results of genetic testing. Ninety-one probands who had not undergone genetic testing were classified into Probands series 1. Probands with spondyloepiphyseal dysplasia accounted for the majority (*n* = 14), followed by probands with OI (*n* = 9). Fourteen probands with suspected GSD had indeterminate clinical diagnoses due to their complex or ambiguous phenotypes. Overall, 54/91 probands were confirmed to have 31 disease-causing genes, for a diagnosis rate of 59%. Among 54 probands with clear molecular diagnoses, lysosomal storage diseases with skeletal involvement were the most common (*n* = 7), followed by OI (*n* = 6) and members of the type 2 collagen group (*n* = 5). In probands with indeterminate clinical diagnoses, 5/14 (36%) had received identified molecular diagnoses, including Larsen syndrome (2 cases), hereditary motor neuropathy (1 case), hypochondroplasia (1 case), and short stature with non-specific skeletal abnormalities (1 case).

Probands series 2 contained 97 probands who had previously received Sanger sequencing with negative results (Sanger sequencing results are shown in Supplementary Table 1). OI was the most common initial diagnosis among the probands (*n* = 47), and no pathogenic variants were found in *COL1A1/2* genes all of them. The next most common initial diagnosis was hypophosphatemic rickets (*n* = 15), and no pathogenic variants were found in *PHEX*. In Probands series 2, 21/97 probands had received confirmed molecular diagnoses, for an overall diagnosis rate of 22%. A total of 7/47 probands suspected with OI carried variants in 6 disease-causing genes, namely, 1 *IFITM5*, 1 *CRTAP*, 2 *BMP1*, 1 *PLS3*, and 1 *COL1A2* (c.432 + 4_432 + 7delAGTA was ignored previously), and 1 case of Camurati-Engelmann disease with pathogenic variants in *TGF*β*-1*, which was misdiagnosed. Heterozygous variants in *COL10A1* gene were detected in 2/15 probands suspected to have hypophosphatemic rickets, and no candidate gene was found in the other 13 probands. The most common molecular diagnosis was OI (*n* = 8). Two probands who were not previously suspected to have OI were included, detected with pathogenic/likely pathogenic variants in *COL1A2* and *IFITM5*.

In Probands series 3, to test the sensitivity of the panel, we included 35 probands who were identified to have clearly pathogenic/likely pathogenic variants. There were 44 variants in 19 genes, namely, 32 single-nucleotide substitution variants in exons, 7 intron boundary variants affecting splicing function, 3 deletions of one to two nucleotides, and 1 insertion/deletion variant. The results of panel testing completely covered 44 reference variants; thus, the sensitivity of panel detection of variants was 100%. In this group, a molecular diagnosis of OI accounted for 37% of probands (*n* = 13), followed by members of the type 2 collagen group in 20% (*n* = 7).

### Coincidence Rate of Clinical Diagnosis and Molecular Diagnosis

Targeted exome sequencing confirmed the molecular diagnoses of 110 probands, 35 of whom had confirmed molecular diagnoses before testing. Of the remaining 75 probands, we found that 19 (25%) had their diagnoses modified after sequencing (Table 3). The misdiagnosis rates of Probands series 1 and 2 were 17% (9/54) and 48% (10/21), respectively. Because of the overlap of clinical manifestations and the heterogeneity of phenotypes, mucopolysaccharidosis and spondylo-epi-metaphyseal dysplasias were difficult to distinguish in some cases. In our study, 4 probands who were misdiagnosed with spondylo-epi-metaphyseal dysplasia or progressive pseudorheumatoid dysplasia were ultimately diagnosed with mucopolysaccharidosis caused by *GALNS*. Three probands who were initially diagnosed with mucopolysaccharidosis ultimately had their diagnoses modified to progressive pseudorheumatoid dysplasia, hypochondroplasia, and Kniest dysplasia. Two probands with metaphyseal chondrodysplasia had been misdiagnosed with hypophosphatemic rickets due to low serum phosphorus levels. Two probands with OI had also been misdiagnosed due to overlapping clinical manifestations with other diseases. Interestingly, one proband (GSD2201) was initially diagnosed with Paget’s disease or progressive diaphyseal dysplasia; eventually, he was found to carry a heterozygous variant in *TGF*β*-2* gene (c.220A>C, p.T74P) that could lead to Loeys-Dietz syndrome 4 (LDS4). To date, only five other centers have reported cases of LDS caused by *TGF*β*-2* (Boileau et al., 2012; Lindsay et al., 2012; Renard et al., 2013; Gago-Díaz et al., 2014; Ritelli et al., 2014), and ours is the first report in China.

**TABLE 3:** Pathogenic variants in the 19 probands with modified molecular diagnoses.

Group	ID	Clinical diagnosis	Molecular diagnosis	Gene	Genomic variant(s)	Segregation
Probands series 1	GSD2378	Multiple epiphyseal dysplasia	Hypochondroplasia	FGFR3	NM_000142, c.1620C>G, p.N540K(het)	*De novo*
	GSD1023	Hypophosphatemic rickets	Metaphyseal chondrodysplasia, Schmid type	COL10A1	NM_000493, c.1783G>C, p.G595R(het)	Maternal
	GSD0001	Hypophosphatemic rickets	Metaphyseal chondrodysplasia, Schmid type	COL10A1	NM_000493, c.1767dupT, p.T590fs(het)	*De novo*
	GSD0526	Progressive pseudorheumatoid dysplasia	Pseudoachondroplasia	COMP	NM_000095, c.1424A>G, p.D475G(het)	*De novo*
	GSD2416	Progressive pseudorheumatoid dysplasia	Mucopolysaccharidosis IVA	GALNS	NM_000512, c.911G>A, p.G304D(hom)	*De novo*
	GSD0373	Fibrodysplasia ossificans progressiva	Osteogenesis imperfecta V	IFITM5	NM_001025295, UTR5, c.-14C>T(het)	*De novo*
	GSD1270	Hajdu-Cheney syndrome	Osteogenesis imperfecta I	COL1A1	NM_000088, c.3595A>G, p.S1199G(het)	Unknown (mother died)
	GSD1563	Progressive pseudorheumatoid dysplasia	Spondyloepiphyseal dysplasia	COL2A1	NM_001844, c.620G>A, p.G207E(het)	*De novo*
	GSD2750	Osteogenesis imperfecta	Camurati-Engelmann disease	TGFβ-1	NM_000660, c.652C>T, p.R218C(het)	*De novo*
	GSD2201	Paget’s disease	Loeys-Dietz syndrome 4	TGFβ-2	NM_001135599, c.220A>C, p.T74P(het)	Unknown (father died)
	GSD2778	Spondyloepiphyseal dysplasia	Stickler syndrome	COL2A1	NM_001844, c.870+5G>A(het)	*De novo*
Probands series 2	GSD0412	Spondyloepiphyseal dysplasia	Brachytelephalangic chondrodysplasia punctata	ARSE	NM_000047, c.217G>A, p.G73S(xl)	*De novo*
	GSD0962	Spondyloepiphyseal dysplasia	Mucopolysaccharidosis IVA	GALNS	NM_000512, c.1156C>T, p.R386C; c.1288C>G, p.H430D(com-het)	One site paternal; one site *de novo*
	GSD2503	Spondylometaphyseal dysplasia	Mucopolysaccharidosis IVA	GALNS	NM_000512, c.1279_1286del, p.V427fs; c.775C > T, p.R259W(com-het)	One site maternal; one site *de novo*
	GSD0153	Mucopolysaccharidosis	Progressive pseudorheumatoid dysplasia	WISP3	NM_198239, c.670dupA, p.W223fs(hom)	One site paternal; one site *de novo*
	GSD0441	Mucopolysaccharidosis	Hypochondroplasia	FGFR3	NM_000142, c.1612A>G, p.I538V(het)	*De novo*
	GSD1185	Mucopolysaccharidosis	Kniest dysplasia	COL2A1	NM_001844, c.654+1G>C(het)	*De novo*
	GSD1556	Spondyloepiphyseal dysplasia	Pseudoachondroplasia	COMP	NM_000095, c.2038A>T, p.K680X(het)	*De novo*
	GSD0842	Spondylometaphyseal dysplasia	Mucopolysaccharidosis IVA	GALNS	NM_000512, c.1498G>T, p.G500C; c.1429_1455del, p.477_485del(com-het)	One site maternal; one site paternal

*het, heterozygous; hom, homozygous; com-het, compound heterozygous; xl, X-linked; del, deletion; dup, duplication.*

### Influence of Testing Results on Probands

For probands whose disease-causing genes were identified, the results of genetic testing improved the subsequent clinical management. Twenty probands avoided unnecessary examinations, 17 probands received new treatment plans according to their molecular diagnosis results, and 6 probands were warned of complications affecting other systems. Additionally, 21 families received genetic counseling. Eight families had the target disease-causing gene(s) of at-risk members tested by Sanger sequencing, which ruled out the possibility of variants. Four families had a prenatal genetic diagnosis to give birth to a healthy baby, and the specific test results are shown in Table 4. Other patients were given symptomatic treatment or comfort care according to their molecular diagnoses. For those probands in whom the known skeletal disease-causing genes had been excluded, we suggested WES for comprehensive genetic exploration. After obtaining the probands’ consent, we performed WES on 10 probands who had no variants of known candidate genes. After analysis, we confirmed the molecular diagnoses of 3/10 probands (Supplementary Table 1).

**TABLE 4:** Prenatal genetic diagnosis in four families with molecular diagnoses.

Pregnant woman	Detected gene	Sanger sequencing results	Inheritance	The final decision of the patient
Proband (GSD0768)	COL2A1	p.Gly204Val (NM_001844.4), het	AD	Terminal pregnancy
Proband’s (GSD2034) mother	COMP	No variant	AD	Pregnancy
Proband’s (GSD2953) mother	GALNS	p.Pro169Leu(NM_000512), het	AR	Pregnancy
Proband (GSD2861)	SLC34A3	p.Pro401Arg(NM_001177316), het	AR	Pregnancy

*Fetal DNA was obtained from amniotic fluid, approved by the Ethics Committee of Shanghai Jiao Tong University Affiliated Sixth People’s Hospital. het, heterozygous; AD, autosomal dominant inheritance; AR, autosomal recessive inheritance.*

### Cost-Efficiency Analysis

We tracked the time from peripheral blood sampling to receiving the test report in 114 probands; these intervals ranged from 16 to 72 days, with a median of 45 days. In our center, the average turnaround time of Sanger sequencing of a single sample was approximately 30 days, and that of WGS ranged from 2 to 4 months. In terms of cost, it costs approximately $130 to run TES of one sample, $50 for Sanger sequencing, and $320 for WES. We did not charge any testing fees to the patients, and all the costs of genetic sequencing were borne by our center. Before TES, the 97 probands in the Sanger sequencing–negative group had spent an average of $81 on Sanger sequencing, but the disease-causing genes were not definitively identified. In general, TES had the best cost–benefit ratio.

## Discussion

In this study, we conducted TES on 223 probands suspected to have GSD; the panel contained 322 known disease-causing genes. Ultimately, we found 129 pathogenic/likely pathogenic variants in 110 probands, 63% of which were missense variants and 50% of which were *de novo*. The overall diagnostic rate of TES was 49% (Probands series 1, 2, and 3 had diagnostic rates of 59, 22, and 100%, respectively). The results of testing helped clinicians correct the original diagnoses of 19 probands, improved clinical management in 37 probands, and guided 20 patients to have genetic counseling. The clinical experience of our center suggests that TES would be a cost-effective option for patients with suspected GSD.

Since genetic medicine has only recently been established in China (Zhang et al., 2011), clinical genetic services have not been promoted (Zhao et al., 2013). Few formally trained physicians work in this field. In addition, GSD has overlapping clinical phenotypes, great genetic heterogeneity and numerous disease-causing genes, all of which increase the difficulty of diagnosis. Most patients have not been properly evaluated and treated, and they must go to a more specialized hospital for a definite diagnosis. It is a waste of time and money for patients to visit doctors repeatedly and undergo repeated examinations. With the continuous innovation of sequencing technology, many sequencing methods have been applied in clinical diagnosis to solve this problem (Rehm et al., 2013). Compared with Sanger sequencing, in which candidate genes must be tested one by one, NGS can greatly shorten the time to diagnosis. TES has a shorter turnaround time and a much lower price than WES or WGS. At our center, WES (including library construction, data analysis, and preservation) costs $320 per sample, and TES costs $130 per sample. In our country, genetic testing is excluded from insurance (Chopra and Duan, 2015), but the cost of TES is affordable to patients. This makes it possible for the panel to be applied for clinical diagnosis.

The overall diagnosis rate of TES was 49%, and the detection rate of known pathogenic variants was 100%. The main mode of inheritance was autosomal dominant (65%). OI (*n* = 70) was the most common disease in our center, with 28/70 of probands having a confirmed molecular diagnosis of OI. Among these 28 probands, 50% (14/28) had variants in *COL1A1/2*. Many studies have shown that 80–90% of OI is caused by *COL1A1/2* (Marini et al., 2017). In this study, 46/70 of the probands had previously undergone Sanger sequencing; thus, the proportion of special types of OI was increased. The diagnosis rate of spondyloepiphyseal dysplasia was 68% (25/37).

In a category of clinically clear and genetically heterogeneous disease, TES is the most efficient option. Some teams have focused on the application of panel testing in patients with skeletal dysplasia (Bae et al., 2016; Freire et al., 2019; Uttarilli et al., 2019). One previous study conducted TES on 185 skeletal dysplasia patients and achieved an overall diagnosis rate of 55%, which was higher than what we achieved at our center (49%) (Bae et al., 2016). This may be because 43% of the probands in our center had previously undergone Sanger sequencing. Zhou et al. (2018) studied 12 families undergoing prenatal diagnosis of skeletal dysplasia using a targeted panel; they found that a targeted skeletal gene panel with a relatively short turnaround time was highly sensitive for prenatal diagnosis and had a high diagnostic rate (83%). The disadvantage was that the number of patients in the cohort was small. At present, there are few studies on the application of TES in GSD, and more clinical trials are needed in the future to verify its effectiveness.

In this study, there were 113 probands in whom no candidate gene was found; OI was the most common condition (*n* = 42) among these probands, followed by spondylo-epi-metaphyseal dysplasias (*n* = 18) and hypophosphatemic rickets (*n* = 17). The reasons for such cases may be as follows: (1) Pathogenic variants outside the panel may have caused the disease. We performed WES on 10/109 probands and identified pathogenic variants in only 3/10 probands. Some probands appeared to carry pathogenic variants for other genetic diseases that were not included in the panel. (2) Probands may have had new, previously unreported causative genes. For example, the most common disease that did not show any sequence variant was OI, which may be due to the existence of some new unknown causative genes that were not included in this panel. By 2019, 20 types of OI had been recognized worldwide, and 18 causative genes had been discovered (Etich et al., 2020). In the past year, studies have successively reported three new causative genes for OI (*MESD*, *KDELR2*, and *CCDC134*) (Dubail et al., 2020; van Dijk et al., 2020; Stürznickel and Jähn-Rickert, 2021), which were not included in the panel. Future studies may identify even more causative genes for OI. (3) The large insertion/deletion variants and chromosomal abnormality could also explain a part of disease. Since most GSDs are not a result of chromosomal abnormalities and large insertion/deletion (Lewiecki et al., 2020), we did not focus on this. For example, various studies have reported that the diagnosis rate of hypophosphatemic rickets is approximately 45–79% (Ruppe et al., 2011; Zhang et al., 2019); however, the detection rate in our center was considerably lower. According to statistics, large insertion/deletion variants account for at least 10% of all variants in the *PHEX* gene (Rowe et al., 1997). At present, many software were used in analyzing the large deletion and duplication in TES data (Bartha and Győrffy, 2019), which may improve the detection rate. Many studies have reported that *PHEX*-MLPA (multiple ligation-dependent probe amplification) increases the detection rate of variants in hypophosphatemic rickets patients (Capelli et al., 2015; Zhang et al., 2019). However, when economic conditions permit, we still recommend WES as the first choice for patients whose pathogenic variants have not been identified.

For patients, a clear molecular diagnosis is of great value in formulating treatment plans, preventing complications, and informing reproductive consultation. To a certain extent, it also alleviates the anxiety of patients who do not sufficiently understand their own diseases. For clinicians, the results of molecular diagnosis can correct an inaccurate clinical diagnosis in a timely manner and prevent the improper treatment of patients. At the same time, we can expand the spectrum of disease phenotypes, summarize genotype–phenotype correlations, and accumulate further diagnostic experience. One informative case is worth mentioning here. Proband GSD2750 was an 11-year-old girl who had initially been diagnosed with OI despite having no family history. She had five fractures before she was 8 years old. She came to the hospital for treatment because of a distal femoral fracture. She had no obvious extraskeletal manifestations, and her vision and hearing were normal. Her blood biochemical examination was normal, and her bone mineral density was low. She had a compression fracture of the lumbar spine and an obvious fracture line of the distal femur, which suggested a diagnosis of OI. After pathogenicity analysis and Sanger sequencing, we identified *TGF*β*-1* as the causative gene. She had a *de novo* p.R218C variant in exon 4, which is a hotspot variant associated with Camurati-Engelmann disease (Van Hul et al., 2019). Retrospectively, we note that we initially focused too much attention on the femoral fracture and low bone mass and ignored the thickening of the femoral cortex. We changed the diagnosis to Camurati-Engelmann disease and reformulated the patient’s treatment.

In summary, we used a targeted panel containing 322 known disease-causing genes to detect 223 probands with suspected GSD. We confirmed the molecular diagnoses of 110 probands, for an overall diagnostic rate of 49%, which is of great significance for the clinical management and genetic counseling of patients with this condition. Although our technique has some limitations, its application value in the diagnosis of GSD cannot be denied. We believe that TES is a cost-effective option for the diagnosis of suspected GSD in countries with limited medical and economic resources. In the future, we hope to gain further clinical experience to illustrate the application value of TES in the diagnosis of GSD.

## Data Availability Statement

The datasets presented in this study can be found in online repositories. The names of the repository/repositories and accession number(s) can be found below: https://doi.org/10.6084/m9.figshare.14695302.v1.

## Ethics Statement

The studies involving human participants were reviewed and approved by the Ethics Committee of Shanghai Jiao Tong University Affiliated Sixth People’s Hospital. Written informed consent to participate in this study was provided by the participants’ legal guardian/next of kin.

## Author Contributions

ZZ and SLi conceived the presented idea, supervised the study, and revised the manuscript. SLv, LX, XL, JZ, WH, and JG contributed to data curation. ZZ, SLi, HY, and CW contributed to funding acquisition. HZ and YH contributed to project administration. SLv wrote the draft manuscript. All authors read and approved the final manuscript.

## Conflict of Interest

The authors declare that the research was conducted in the absence of any commercial or financial relationships that could be construed as a potential conflict of interest.

## Publisher’s Note

All claims expressed in this article are solely those of the authors and do not necessarily represent those of their affiliated organizations, or those of the publisher, the editors and the reviewers. Any product that may be evaluated in this article, or claim that may be made by its manufacturer, is not guaranteed or endorsed by the publisher.

## Supplementary Material

Supplementary Table 1 Demographic characteristics and variants information of 223 probands.

Supplementary Table 2 The list of all the relevant genes in skeletal dysplasia panel.

Supplementary Table 3 Quality metrics of targeted panel sequencing.
