# Supplementary material for: Genetics Evaluation of Targeted Exome Sequencing in 223 Chinese Probands With Genetic Skeletal Dysplasias
Source: Front Cell Dev Biol. 2021 Sep 7;9:715042. doi: 10.3389/fcell.2021.715042 (PMC8452955; doi:10.3389/fcell.2021.715042)
Supplement: Supplementary Table 2 — The list of all the relevant genes in skeletal dysplasia panel. [file Table_2.DOCX]

| Supplementary Table S2. The list of all the relevant genes in skeletal dysplasia panel | | | | |
| --- | --- | --- | --- | --- |
| Target ID | OMIM | Interval | Size | Protein |
| ACAN | 155760 | chr15:89346649-89418610 | 9740 | AGGRECAN |
| ACP5 | 171640 | chr19:11685450-11689826 | 2160 | PHOSPHATASE, ACID, TYPE 5, TARTRATE-RESISTANT |
| ACVR1 | 102576 | chr2:158592933-158732399 | 3712 | ACTIVIN A RECEPTOR, TYPE I |
| ADAMTS10 | 608990 | chr19:8645099-8675645 | 5664 | A DISINTEGRIN-LIKE AND METALLOPROTEINASE WITH THROMBOSPONDIN TYPE 1 MOTIF, 10 |
| ADAMTS17 | 607511 | chr15:100511618-100882208 | 7431 | A DISINTEGRIN-LIKE AND METALLOPROTEINASE WITH THROMBOSPONDIN TYPE 1 MOTIF, 17 |
| ADAMTSL2 | 612277 | chr9:136397261-136440666 | 5133 | ADAMTS-LIKE PROTEIN 2 |
| AGA | 613228 | chr4:178351904-178363682 | 2552 | ASPARTYLGLUCOSAMINIDASE |
| AGPS | 603051 | chr2:178257446-178408589 | 8665 | ALKYLGLYCERONE-PHOSPHATE SYNTHASE |
| AKT1 | 164730 | chr14:105235662-105262105 | 3998 | AKT SERINE/THREONINE KINASE 1 |
| ALPL | 171760 | chr1:21835826-21904930 | 3203 | ALKALINE PHOSPHATASE |
| ALX1 | 601527 | chr12:85674011-85695586 | 1493 | ARISTALESS-LIKE HOMEOBOX 1 |
| ALX3 | 606014 | chr1:110602972-110613347 | 1678 | ARISTALESS-LIKE HOMEOBOX 3 |
| ALX4 | 605420 | chr11:44282253-44331741 | 5666 | ARISTALESS HOMEOBOX 4 |
| AMER1 | 300647 | chrX:63404972-63425649 | 8543 | APC MEMBRANE RECRUITMENT PROTEIN 1 |
| ANKH | 605145 | chr5:14704884-14871912 | 8807 | ANKH INORGANIC PYROPHOSPHATE TRANSPORT REGULATOR |
| ANO5 | 608662 | chr11:22214697-22304938 | 7761 | ANOCTAMIN 5 |
| ANTXR2 | 608041 | chr4:80822746-80994651 | 9133 | ANTHRAX TOXIN RECEPTOR 2 |
| ARHGAP31 | 610911 | chr3:119013195-119138348 | 8679 | RHO GTPase-ACTIVATING PROTEIN 31 |
| ARSB | 611542 | chr5:78073012-78282382 | 7125 | ARYLSULFATASE B |
| ARSE | 300180 | chrX:2852648-2886376 | 3318 | ARYLSULFATASE E |
| ATP6V0A2 | 611716 | chr12:124196840-124246326 | 7541 | ATPase, H+ TRANSPORTING, LYSOSOMAL, V0 SUBUNIT A2 |
| B3GALT6 | 615291 | chr1:1167604-1170445 | 2842 | BETA-1,3-GALACTOSYLTRANSFERASE 6 |
| B4GALT7 | 604327 | chr5:177027094-177037371 | 2027 | BETA-1,4-GALACTOSYLTRANSFERASE 7 |
| BHLHA9 | 615416 | chr17:1173833-1174590 | 758 | BASIC HELIX-LOOP-HELIX FAMILY, MEMBER A9 |
| BMP1 | 112264 | chr8:22022628-22069865 | 5279 | BONE MORPHOGENETIC PROTEIN 1 |
| BMP2 | 112261 | chr20:6748720-6760950 | 3315 | BONE MORPHOGENETIC PROTEIN 2 |
| BMPER | 608699 | chr7:33944498-34195509 | 5831 | BONE MORPHOGENETIC PROTEIN-BINDING ENDOTHELIAL REGULATOR PROTEIN |
| BMPR1B | 603248 | chr4:95679103-96079626 | 6744 | BONE MORPHOGENETIC PROTEIN RECEPTOR, TYPE IB |
| CA2 | 611492 | chr8:86376106-86393746 | 2016 | CARBONIC ANHYDRASE II |
| CANT1 | 613165 | chr17:76987773-77005924 | 3924 | CALCIUM-ACTIVATED NUCLEOTIDASE 1 |
| CASR | 601199 | chr3:121902505-122005369 | 5541 | CALCIUM-SENSING RECEPTOR |
| CC2D2A | 612013 | chr4:15471464-15603205 | 8413 | COILED-COIL AND C2 DOMAINS-CONTAINING PROTEIN 2A |
| CCDC8 | 614145 | chr19:46913561-46916944 | 3384 | COILED-COIL DOMAIN-CONTAINING PROTEIN 8 |
| CDC6 | 602627 | chr17:38444121-38459438 | 3653 | CELL DIVISION CYCLE 6 |
| CDH3 | 114021 | chr16:68678714-68732995 | 4599 | CADHERIN 3 |
| CDKN1C | 600856 | chr11:2904423-2907020 | 2080 | CYCLIN-DEPENDENT KINASE INHIBITOR 1C |
| CDT1 | 605525 | chr16:88870161-88875691 | 3175 | CHROMATIN LICENSING AND DNA REPLICATION FACTOR 1 |
| CEP290 | 610142 | chr12:88442765-88536018 | 10655 | CENTROSOMAL PROTEIN, 290-KD |
| CHST14 | 608429 | chr15:40763135-40765382 | 2248 | CARBOHYDRATE SULFOTRANSFERASE 14 |
| CHST3 | 603799 | chr10:73724095-73773347 | 7123 | CARBOHYDRATE SULFOTRANSFERASE 3 |
| CHSY1 | 608183 | chr15:101715903-101792162 | 4700 | CHONDROITIN SULFATE SYNTHASE 1 |
| CKAP2L | 616174 | chr2:113493902-113522279 | 5240 | CYTOSKELETON-ASSOCIATED PROTEIN 2-LIKE |
| CKAP4 | 618595 | chr12:106631634-106641738 | 3136 | CYTOSKELETON-ASSOCIATED PROTEIN 4 |
| CLCN5 | 300008 | chrX:49687200-49863917 | 11482 | CHLORIDE CHANNEL 5 |
| CLCN7 | 602727 | chr16:1494909-1525110 | 5476 | CHLORIDE CHANNEL 7 |
| COG1 | 606973 | chr17:71189148-71204670 | 3734 | COMPONENT OF OLIGOMERIC GOLGI COMPLEX 1 |
| COL10A1 | 120110 | chr6:116440060-116447321 | 3440 | COLLAGEN, TYPE X, ALPHA-1 |
| COL11A1 | 120280 | chr1:103341998-103574077 | 10910 | COLLAGEN, TYPE XI, ALPHA-1 |
| COL11A2 | 120290 | chr6:33130444-33160270 | 9907 | COLLAGEN, TYPE XI, ALPHA-2 |
| COL1A1 | 120150 | chr17:48261432-48279025 | 8477 | COLLAGEN, TYPE I, ALPHA-1 |
| COL1A2 | 120160 | chr7:94023848-94060569 | 8011 | COLLAGEN, TYPE I, ALPHA-2 |
| COL2A1 | 120140 | chr12:48366723-48398310 | 7787 | COLLAGEN, TYPE II, ALPHA-1 |
| COL9A1 | 120210 | chr6:70925718-71012811 | 5960 | COLLAGEN, TYPE IX, ALPHA-1 |
| COL9A2 | 120260 | chr1:40766138-40782964 | 4431 | COLLAGEN, TYPE IX, ALPHA-2 |
| COL9A3 | 120270 | chr20:61448389-61472536 | 4085 | COLLAGEN, TYPE IX, ALPHA-3 |
| COMP | 600310 | chr19:18893558-18902139 | 3402 | CARTILAGE OLIGOMERIC MATRIX PROTEIN |
| CREB3L1 | 616215 | chr11:46299164-46342997 | 3310 | cAMP RESPONSE ELEMENT-BINDING PROTEIN 3-LIKE 1 |
| CREBBP | 600140 | chr16:3775031-3930146 | 11746 | CREB-BINDING PROTEIN |
| CRTAP | 605497 | chr3:33155425-33189290 | 7001 | CARTILAGE-ASSOCIATED PROTEIN |
| CTGF | 121009 | chr6:132269292-132272543 | 2593 | CONNECTIVE TISSUE GROWTH FACTOR |
| CTSA | 613111 | chr20:44518758-44527483 | 3851 | CATHEPSIN A |
| CTSK | 601105 | chr1:150768659-150780942 | 2210 | CATHEPSIN K |
| CUL7 | 609577 | chr6:43005330-43021708 | 6896 | CULLIN 7 |
| DDR2 | 191311 | chr1:162602203-162750272 | 4194 | DISCOIDIN DOMAIN RECEPTOR FAMILY, MEMBER 2 |
| DHCR24 | 606418 | chr1:55315275-55352946 | 4736 | 24-DEHYDROCHOLESTEROL REDUCTASE |
| DHODH | 126064 | chr16:72042618-72059341 | 2877 | DIHYDROOROTATE DEHYDROGENASE |
| DLL3 | 602768 | chr19:39989532-39999146 | 2783 | DELTA-LIKE CANONICAL NOTCH LIGAND 3 |
| DLX3 | 600525 | chr17:48067344-48072613 | 2752 | DISTAL-LESS HOMEOBOX 3 |
| DLX5 | 600028 | chr7:96649677-96654168 | 1574 | DISTAL-LESS HOMEOBOX 5 |
| DLX6 | 600030 | chr7:96635265-96640377 | 2025 | DISTAL-LESS HOMEOBOX 6 |
| DMP1 | 600980 | chr4:88571429-88585537 | 2986 | DENTIN MATRIX ACIDIC PHOSPHOPROTEIN 1 |
| DOCK6 | 614194 | chr19:11309944-11373193 | 8800 | DEDICATOR OF CYTOKINESIS 6 |
| DVL1 | 601365 | chr1:1270633-1284517 | 3674 | DISHEVELLED 1 |
| DYM | 607461 | chr18:46570147-46987104 | 3478 | DYMECLIN |
| DYNC2H1 | 603297 | chr11:102980135-103350616 | 18199 | DYNEIN, CYTOPLASMIC 2, HEAVY CHAIN 1 |
| EBP | 300205 | chrX:48380139-48387129 | 1407 | EMOPAMIL-BINDING PROTEIN |
| EFNB1 | 300035 | chrX:68048815-68062031 | 3580 | EPHRIN B1 |
| EFTUD2 | 603892 | chr17:42927630-42977018 | 5961 | ELONGATION FACTOR Tu GTP-BINDING DOMAIN-CONTAINING 2 |
| EIF2AK3 | 604032 | chr2:88856233-88927119 | 5733 | EUKARYOTIC TRANSLATION INITIATION FACTOR 2-ALPHA KINASE 3 |
| ENPP1 | 173335 | chr6:132129131-132216320 | 8692 | ECTONUCLEOTIDE PYROPHOSPHATASE/PHOSPHODIESTERASE 1 |
| EOGT | 614789 | chr3:69024338-69063070 | 5566 | EGF DOMAIN-SPECIFIC O-LINKED N-ACETYLGLUCOSAMINE TRANSFERASE |
| EP300 | 602700 | chr22:41488589-41576106 | 10311 | E1A-BINDING PROTEIN, 300-KD |
| ERF | 611888 | chr19:42751688-42759341 | 3173 | ETS2 REPRESSOR FACTOR |
| ESCO2 | 609353 | chr8:27632033-27662449 | 3908 | ESTABLISHMENT OF SISTER CHROMATID COHESION N-ACEYTLTRANSFERASE 2 |
| EVC | 604831 | chr4:5712899-5816056 | 7761 | EVC CILIARY COMPLEX SUBUNIT 1 |
| EVC2 | 607261 | chr4:5564121-5711300 | 6280 | EVC CILIARY COMPLEX SUBUNIT 2 |
| EXT1 | 608177 | chr8:118811577-119124083 | 3913 | EXOSTOSIN GLYCOSYLTRANSFERASE 1 |
| EXT2 | 608210 | chr11:44117074-44267005 | 4652 | EXOSTOSIN GLYCOSYLTRANSFERASE 2 |
| EZH2 | 601573 | chr7:148504439-148581466 | 4086 | ENHANCER OF ZESTE 2 POLYCOMB REPRESSIVE COMPLEX 2 SUBUNIT |
| FAM111A | 615292 | chr11:58910194-58922536 | 4682 | FAMILY WITH SEQUENCE SIMILARITY 111, MEMBER A |
| FAM20C | 611061 | chr7:192944-300765 | 3280 | FAMILY WITH SEQUENCE SIMILARITY 20, MEMBER C |
| FAM58A | 300708 | chrX:152853358-152864657 | 1538 | FAMILY WITH SEQUENCE SIMILARITY 58, MEMBER A |
| FBLN1 | 135820 | chr22:45898694-45997039 | 5857 | FIBULIN 1 |
| FBN1 | 134797 | chr15:48700478-48938010 | 14995 | FIBRILLIN 1 |
| FBN2 | 612570 | chr5:127593576-127873760 | 13974 | FIBRILLIN 2 |
| FERMT3 | 607901 | chr11:63974127-63991388 | 3302 | FERMITIN FAMILY, MEMBER 3 |
| FGF10 | 602115 | chr5:44305072-44388809 | 777 | FIBROBLAST GROWTH FACTOR 10 |
| FGF16 | 300827 | chrX:76709622-76712038 | 451 | FIBROBLAST GROWTH FACTOR 16 |
| FGF23 | 605380 | chr12:4477368-4488919 | 3168 | FIBROBLAST GROWTH FACTOR 23 |
| FGF9 | 600921 | chr13:22245190-22278665 | 4680 | FIBROBLAST GROWTH FACTOR 9 |
| FGFR1 | 136350 | chr8:38268631-38326377 | 7353 | FIBROBLAST GROWTH FACTOR RECEPTOR 1 |
| FGFR2 | 176943 | chr10:123237819-123357997 | 6623 | FIBROBLAST GROWTH FACTOR RECEPTOR 2 |
| FGFR3 | 134934 | chr4:1795014-1810624 | 5388 | FIBROBLAST GROWTH FACTOR RECEPTOR 3 |
| FIG4 | 609390 | chr6:110012399-110146659 | 4255 | FIG4 PHOSPHOINOSITIDE 5-PHOSPHATASE |
| FKBP10 | 607063 | chr17:39968937-39979494 | 3383 | FK506-BINDING PROTEIN 10 |
| FLNA | 300017 | chrX:153576875-153603031 | 10910 | FILAMIN A |
| FLNB | 603381 | chr3:57994102-58158007 | 11910 | FILAMIN B |
| FMN1 | 136535 | chr15:33057720-33486959 | 17798 | FORMIN 1 |
| FUCA1 | 612280 | chr1:24171547-24194884 | 2517 | FUCOSIDASE, ALPHA-L |
| FZD2 | FZD2 | chr17:42634787-42638655 | 3869 | FRIZZLED CLASS RECEPTOR 2 |
| GALNS | 612222 | chr16:88880117-88923399 | 3063 | GALACTOSAMINE-6-SULFATE SULFATASE |
| GALNT3 | 601756 | chr2:166604288-166650828 | 3817 | UDP-N-ACETYL-ALPHA-D-GALACTOSAMINE:POLYPEPTIDE N-ACETYLGALACTOSAMINYLTRANSFERASE 3 |
| GDF3 | 606522 | chr12:7842356-7848385 | 1324 | GROWTH/DIFFERENTIATION FACTOR 3 |
| GDF5 | 601146 | chr20:34021120-34042593 | 2793 | GROWTH/DIFFERENTIATION FACTOR 5 |
| GDF6 | 601147 | chr8:97154533-97173045 | 3801 | GROWTH/DIFFERENTIATION FACTOR 6 |
| GJA1 | 121014 | chr6:121756698-121770915 | 3269 | GAP JUNCTION PROTEIN, ALPHA-1 |
| GLB1 | 611458 | chr3:33038075-33138747 | 3774 | GALACTOSIDASE, BETA-1 |
| GLI3 | 165240 | chr7:42000523-42276643 | 8964 | GLI-KRUPPEL FAMILY MEMBER 3 |
| GNAS | 139320 | chr20:57414748-57486276 | 8265 | GNAS COMPLEX LOCUS |
| GNPTAB | 607840 | chr12:102139250-102224670 | 6680 | N-ACETYLGLUCOSAMINE-1-PHOSPHOTRANSFERASE, ALPHA/BETA SUBUNITS |
| GNPTG | 607838 | chr16:1401875-1413377 | 1795 | N-ACETYLGLUCOSAMINE-1-PHOSPHOTRANSFERASE, GAMMA SUBUNIT |
| GNS | 607664 | chr12:65107197-65153251 | 5828 | N-ACETYLGLUCOSAMINE-6-SULFATASE |
| GORAB | 607983 | chr1:170501238-170522999 | 3780 | GOLGIN, RAB6-INTERACTING |
| GPC6 | 604404 | chr13:93879053-95060298 | 7552 | GLYPICAN 6 |
| GPX4 | 138322 | chr19:1103900-1106813 | 1631 | GLUTATHIONE PEROXIDASE 4 |
| GREM1 | 603054 | chr15:33010180-33026895 | 4238 | GREMLIN 1 HOMOLOG, CYSTINE KNOT SUPERFAMILY |
| GUSB | 611499 | chr7:65425647-65447326 | 2899 | BETA-GLUCURONIDASE |
| HDAC4 | 605314 | chr2:239969839-240322668 | 10326 | HISTONE DEACETYLASE 4 |
| HDAC8 | 300269 | chrX:71549341-71792978 | 4235 | HISTONE DEACETYLASE 8 |
| HES7 | 608059 | chr17:8023883-8027435 | 1882 | HES FAMILY bHLH TRANSCRIPTION FACTOR 7 |
| HGSNAT | 610453 | chr8:42995567-43057995 | 6114 | HEPARAN-ALPHA-GLUCOSAMINIDE N-ACETYLTRANSFERASE |
| HOXA11 | 142958 | chr7:27220751-27224860 | 2753 | HOMEOBOX A11 |
| HOXA13 | 142959 | chr7:27236474-27239750 | 2614 | HOMEOBOX A13 |
| HOXD13 | 142989 | chr2:176957507-176960691 | 2427 | HOMEOBOX D13 |
| HPGD | 601688 | chr4:175411303-175444074 | 3377 | 15-HYDROXYPROSTAGLANDIN DEHYDROGENASE |
| HSPG2 | 142461 | chr1:22148700-22263815 | 19193 | HEPARAN SULFATE PROTEOGLYCAN OF BASEMENT MEMBRANE |
| ICK | 612325 | chr6:52866073-52926625 | 6988 | INTESTINAL CELL KINASE |
| IDH1 | 147700 | chr2:209100926-209119892 | 3370 | ISOCITRATE DEHYDROGENASE 1 |
| IDH2 | 147650 | chr15:90627186-90645811 | 2456 | ISOCITRATE DEHYDROGENASE 2 |
| IDS | 300823 | chrX:148558496-148586909 | 8471 | IDURONATE 2-SULFATASE |
| IDUA | 252800 | chr4:980760-998370 | 2918 | ALPHA-L-IDURONIDASE |
| IFITM5 | 614757 | chr11:298176-299551 | 835 | INTERFERON-INDUCED TRANSMEMBRANE PROTEIN 5 |
| IFT122 | 606045 | chr3:129158854-129239375 | 6170 | INTRAFLAGELLAR TRANSPORT 122 |
| IFT140 | 614620 | chr16:1560403-1662134 | 6818 | INTRAFLAGELLAR TRANSPORT 140 |
| IFT172 | 607386 | chr2:27667215-27712703 | 7867 | INTRAFLAGELLAR TRANSPORT 172 |
| IFT43 | 614068 | chr14:76452071-76550441 | 2354 | INTRAFLAGELLAR TRANSPORT 43 |
| IFT80 | 611177 | chr3:159974749-160117345 | 5844 | INTRAFLAGELLAR TRANSPORT 80 |
| IHH | 600726 | chr2:219919117-219925263 | 2222 | INDIAN HEDGEHOG SIGNALING MOLECULE |
| IKBKG | 300248 | chrX:153770434-153793286 | 3311 | INHIBITOR OF NUCLEAR FACTOR KAPPA-B KINASE, REGULATORY SUBUNIT GAMMA |
| IL1RN | 147679 | chr2:113875445-113891618 | 2463 | INTERLEUKIN 1 RECEPTOR ANTAGONIST |
| IMPAD1 | 614010 | chr8:57870463-57906455 | 7480 | INOSITOL MONOPHOSPHATASE DOMAIN-CONTAINING PROTEIN 1 |
| INPPL1 | 600829 | chr11:71935857-71950213 | 6115 | INOSITOL POLYPHOSPHATE PHOSPHATASE-LIKE 1 |
| KAT6B | 605880 | chr10:76586146-76792405 | 9399 | LYSINE ACETYLTRANSFERASE 6B |
| KIF22 | 603213 | chr16:29802009-29816731 | 3223 | KINESIN FAMILY MEMBER 22 |
| KIF7 | 611254 | chr15:90171176-90198707 | 5508 | KINESIN FAMILY MEMBER 7 |
| LBR | 600024 | chr1:225589179-225616582 | 4723 | LAMIN B RECEPTOR |
| LEMD3 | 607844 | chr12:65563326-65642166 | 5448 | LEM DOMAIN-CONTAINING PROTEIN 3 |
| LEPRE1 | 610339 | chr1:43212343-43232667 | 3321 | LEUCINE- AND PROLINE-ENRICHED PROTEOGLYCAN 1 |
| LFNG | 602576 | chr7:2552138-2568835 | 3805 | LFNG O-FUCOSYLPEPTIDE 3-BETA-N-ACETYLGLUCOSAMINYLTRANSFERASE |
| LIFR | 151443 | chr5:38475040-38595532 | 11453 | LEUKEMIA INHIBITORY FACTOR RECEPTOR |
| LMBR1 | 605522 | chr7:156473545-156685927 | 5740 | LIMB DEVELOPMENT MEMBRANE PROTEIN 1 |
| LMNA | 150330 | chr1:156052312-156109905 | 4954 | LAMIN A/C |
| LMX1B | 602575 | chr9:129376697-129463336 | 6230 | LIM HOMEOBOX TRANSCRIPTION FACTOR 1, BETA |
| LONP1 | 605490 | chr19:5691820-5720488 | 4298 | LON PEPTIDASE 1, MITOCHONDRIAL |
| LPIN2 | 605519 | chr18:2916967-3011970 | 7229 | LIPIN 2 |
| LRP4 | 604270 | chr11:46878243-46940198 | 10127 | LOW DENSITY LIPOPROTEIN RECEPTOR-RELATED PROTEIN 4 |
| LRP5 | 603506 | chr11:68080052-68216768 | 6553 | LOW DENSITY LIPOPROTEIN RECEPTOR-RELATED PROTEIN 5 |
| LTBP2 | 602091 | chr14:74964861-75079059 | 10354 | LATENT TRANSFORMING GROWTH FACTOR-BETA-BINDING PROTEIN 2 |
| LTBP3 | 602090 | chr11:65306005-65325724 | 6102 | LATENT TRANSFORMING GROWTH FACTOR-BETA-BINDING PROTEIN 3 |
| MAFB | 608968 | chr20:39314463-39317905 | 3443 | MAF bZIP TRANSCRIPTION FACTOR B |
| MAN2B1 | 609458 | chr19:12757297-12777616 | 4424 | MANNOSIDASE, ALPHA, CLASS 2B, MEMBER 1 |
| MAN2C1 | 154580 | chr15:75648108-75660993 | 4632 | MANNOSIDASE, ALPHA, CLASS 2C, MEMBER 1 |
| MANBA | 609489 | chr4:103552618-103682176 | 4161 | MANNOSIDASE, BETA A, LYSOSOMAL |
| MATN3 | 602109 | chr2:20191788-20212480 | 2983 | MATRILIN 3 |
| MEGF8 | 604267 | chr19:42829736-42882946 | 13267 | MULTIPLE EPIDERMAL GROWTH FACTOR-LIKE DOMAINS 8 |
| MEOX1 | 600147 | chr17:41717733-41739287 | 2538 | MESENCHYME HOMEOBOX 1 |
| MESP2 | 605195 | chr15:90319564-90322007 | 1711 | MESODERM POSTERIOR BASIC HELIX-LOOP-HELIX TRANSCRIPTION FACTOR 2 |
| MGP | 154870 | chr12:15034090-15038878 | 1723 | MATRIX GAMMA-CARBOXYGLUTAMIC ACID |
| MKS1 | 609883 | chr17:56282772-56296991 | 3492 | MKS1 TRANSITION ZONE COMPLEX SUBUNIT 1 |
| MMP13 | 600108 | chr11:102813696-102826488 | 3219 | MATRIX METALLOPROTEINASE 13 |
| MMP2 | 120360 | chr16:55512717-55540628 | 4974 | MATRIX METALLOPROTEINASE 2 |
| MMP9 | 120361 | chr20:44637522-44645225 | 2986 | MATRIX METALLOPROTEINASE 9 |
| MNX1 | 142994 | chr7:156797522-156803372 | 2814 | MOTOR NEURON AND PANCREAS HOMEOBOX 1 |
| MSX2 | 123101 | chr5:174151550-174157927 | 2308 | MSH HOMEOBOX 2 |
| MYCN | 164840 | chr2:16080535-16087154 | 3188 | MYCN PROTOONCOGENE, bHLH TRANSCRIPTION FACTOR |
| NAGLU | 609701 | chr17:40687926-40696491 | 3082 | N-ACETYLGLUCOSAMINIDASE, ALPHA- |
| NEK1 | 604588 | chr4:170314396-170533803 | 7507 | NEVER IN MITOSIS GENE A-RELATED KINASE 1 |
| NEU1 | 608272 | chr6:31826804-31830734 | 2371 | NEURAMINIDASE 1 |
| NF1 | 613113 | chr17:29421920-29704720 | 16160 | NEUROFIBROMIN 1 |
| NFIX | 164005 | chr19:13106559-13209635 | 6542 | NUCLEAR FACTOR I/X |
| NIPBL | 608667 | chr5:36876836-37065946 | 12689 | NIPPED-B-LIKE |
| NKX3-2 | 602183 | chr4:13542429-13546139 | 2341 | NK3 HOMEOBOX 2 |
| NLRP3 | 606416 | chr1:247579433-247612431 | 4995 | NLR FAMILY, PYRIN DOMAIN-CONTAINING 3 |
| NOG | 602991 | chr17:54671035-54672976 | 1942 | NOGGIN |
| NOTCH2 | 600275 | chr1:120454151-120612342 | 13525 | NOTCH RECEPTOR 2 |
| NPPC | 600296 | chr2:232786779-232791138 | 927 | NATRIURETIC PEPTIDE PRECURSOR C |
| NPR2 | 108961 | chr9:35792381-35809753 | 4530 | NATRIURETIC PEPTIDE RECEPTOR 2 |
| NSD1 | 606681 | chr5:176560055-176727239 | 14259 | NUCLEAR RECEPTOR-BINDING SET DOMAIN PROTEIN 1 |
| NSDHL | 300275 | chrX:151999486-152037932 | 2080 | NAD(P)H STEROID DEHYDROGENASE-LIKE PROTEIN |
| OBSL1 | 610991 | chr2:220415425-220436293 | 8375 | OBSCURIN-LIKE 1 |
| OFD1 | 300170 | chrX:13752807-13787505 | 4801 | OFD1 CENTRIOLE AND CENTRIOLAR SATELLITE PROTEIN |
| ORC1 | 601902 | chr1:52838476-52870168 | 4019 | ORIGIN RECOGNITION COMPLEX, SUBUNIT 1 |
| ORC4 | 603056 | chr2:148687941-148779198 | 7839 | ORIGIN RECOGNITION COMPLEX, SUBUNIT 4 |
| ORC6 | 607213 | chr16:46723533-46732331 | 2037 | ORIGIN RECOGNITION COMPLEX, SUBUNIT 6 |
| OSTM1 | 607649 | chr6:108362588-108395966 | 4767 | OSTEOPETROSIS-ASSOCIATED TRANSMEMBRANE PROTEIN 1 |
| P4HB | 176790 | chr17:79801009-79818569 | 3128 | PROCOLLAGEN-PROLINE, 2-OXOGLUTARATE-4-DIOXYGENASE, BETA SUBUNIT |
| PAM16 | 614336 | chr16:4390227-4401398 | 834 | PRESEQUENCE TRANSLOCASE-ASSOCIATED MOTOR 16 |
| PAPSS2 | 603005 | chr10:89419451-89507487 | 4508 | 3-PRIME-PHOSPHOADENOSINE 5-PRIME-PHOSPHOSULFATE SYNTHASE 2 |
| PCNT | 605925 | chr21:47744011-47865707 | 13283 | PERICENTRIN |
| PCYT1A | 123695 | chr3:195964591-196014648 | 2759 | PHOSPHATE CYTIDYLYLTRANSFERASE 1, CHOLINE, ALPHA ISOFORM |
| PDE3A | 123805 | chr12:20522154-20837066 | 8460 | PHOSPHODIESTERASE 3A |
| PDE4D | 600129 | chr5:58264841-59783950 | 12342 | PHOSPHODIESTERASE 4D |
| PEX7 | 601757 | chr6:137143677-137235097 | 1982 | PEROXISOME BIOGENESIS FACTOR 7 |
| PHEX | 300550 | chrX:22050537-22269452 | 7269 | PHOSPHATE-REGULATING ENDOPEPTIDASE HOMOLOG, X-LINKED |
| PIGV | 610274 | chr1:27114429-27124919 | 3155 | PHOSPHATIDYLINOSITOL GLYCAN ANCHOR BIOSYNTHESIS CLASS V PROTEIN |
| PIK3CA | 171834 | chr3:178866286-178952522 | 4759 | PHOSPHATIDYLINOSITOL 3-KINASE, CATALYTIC, ALPHA |
| PITX1 | 602149 | chr5:134363399-134369989 | 2533 | PAIRED-LIKE HOMEODOMAIN TRANSCRIPTION FACTOR 1 |
| PLEKHM1 | 611466 | chr17:43513241-43568171 | 5899 | PLECKSTRIN HOMOLOGY DOMAIN-CONTAINING PROTEIN, FAMILY M, MEMBER 1 |
| PLOD2 | 601865 | chr3:145787203-145879307 | 5059 | PROCOLLAGEN-LYSINE, 2-OXOGLUTARATE 5-DIOXYGENASE 2 |
| PLS3 | 300131 | chrX:114795152-114885204 | 5264 | PLASTIN 3 |
| POLR1C | 610060 | chr6:43484737-43497139 | 1981 | POLYMERASE I, RNA, SUBUNIT C |
| POLR1D | 613715 | chr13:28194855-28241584 | 3270 | POLYMERASE I, RNA, SUBUNIT D |
| POP1 | 602486 | chr8:99129496-99172094 | 5621 | POP1 HOMOLOG, RIBONUCLEASE P/MRP SUBUNIT |
| POR | 124015 | chr7:75544395-75616198 | 3299 | CYTOCHROME P450 OXIDOREDUCTASE |
| PPIB | 123841 | chr15:64447989-64455379 | 1278 | PEPTIDYL-PROLYL ISOMERASE B |
| PRKAR1A | 188830 | chr17:66409739-66547482 | 5985 | PROTEIN KINASE, cAMP-DEPENDENT, REGULATORY, TYPE I, ALPHA |
| PTDSS1 | 612792 | chr8:97274089-97346804 | 3212 | PHOSPHATIDYLSERINE SYNTHASE 1 |
| PTH1R | 168468 | chr3:46919211-46945314 | 2996 | PARATHYROID HORMONE 1 RECEPTOR |
| PTHLH | 168470 | chr12:28110992-28124941 | 2939 | PARATHYROID HORMONE-LIKE HORMONE |
| PTPN11 | 176876 | chr12:112856511-112947742 | 7377 | PROTEIN-TYROSINE PHOSPHATASE, NONRECEPTOR-TYPE, 11 |
| PYCR1 | 179035 | chr17:79890237-79895229 | 2652 | PYRROLINE-5-CARBOXYLATE REDUCTASE 1 |
| RAB23 | 606144 | chr6:57051766-57087137 | 5379 | RAS-ASSOCIATED PROTEIN RAB23 |
| RAB33B | 605950 | chr4:140374936-140397094 | 3968 | RAS-ASSOCIATED PROTEIN RAB33B |
| RAD21 | 606462 | chr8:117858148-117887130 | 4450 | RAD21 COHESIN COMPLEX COMPONENT |
| RASGRP2 | 605577 | chr11:64494358-64512953 | 3532 | RAS GUANYL NUCLEOTIDE-RELEASING PROTEIN 2 |
| RBM8A | 605313 | chr1:145507532-145513560 | 5259 | RNA-BINDING MOTIF PROTEIN 8A |
| RBPJ | 147183 | chr4:26321307-26436777 | 6928 | RECOMBINATION SIGNAL-BINDING PROTEIN FOR IMMUNOGLOBULIN KAPPA J REGION |
| RECQL4 | 603780 | chr8:145736642-145743235 | 4867 | RECQ PROTEIN-LIKE 4 |
| RMRP | 157660 | chr9:35657723-35658040 | 318 | MITOCHONDRIAL RNA-PROCESSING ENDORIBONUCLEASE, RNA COMPONENT OF |
| RNU4ATAC | 601428 | chr2:122288431-122288610 | 180 | RNA, U4ATAC SMALL NUCLEAR |
| ROR2 | 602337 | chr9:944848  53-94712469 | 4641 | RECEPTOR TYROSINE KINASE-LIKE ORPHAN RECEPTOR 2 |
| RPGRIP1L | 610937 | chr16:53633126-53737871 | 8274 | RPGRIP1-LIKE |
| RUNX2 | 600211 | chr6:45296029-45518844 | 6269 | RUNT-RELATED TRANSCRIPTION FACTOR 2 |
| SALL1 | 602218 | chr16:51169861-51185208 | 5564 | SAL-LIKE 1 |
| SALL4 | 607343 | chr20:50399231-50419087 | 5025 | SAL-LIKE 4 |
| SBDS | 607444 | chr7:66452665-66460613 | 1855 | SBDS PROTEIN |
| SEC24D | 607186 | chr4:119643953-119757376 | 5241 | SEC24-RELATED GENE FAMILY, MEMBER D |
| SERPINF1 | 172860 | chr17:1665234-1680884 | 1933 | SERPIN PEPTIDASE INHIBITOR, CLADE F, MEMBER 1 |
| SERPINH1 | 600943 | chr11:75273076-75283874 | 2620 | SERPIN PEPTIDASE INHIBITOR, CLADE H, MEMBER 1 |
| SETD2 | 612778 | chr3:47057873-47205492 | 9483 | SET DOMAIN-CONTAINING PROTEIN 2 |
| SF3B4 | 605593 | chr1:149895184-149900169 | 2293 | SPLICING FACTOR 3B, SUBUNIT 4 |
| SGSH | 605270 | chr17:78183054-78194224 | 3168 | N-SULFOGLUCOSAMINE SULFOHYDROLASE |
| SH3BP2 | 602104 | chr4:2794725-2842848 | 10590 | SH3 DOMAIN-BINDING PROTEIN 2 |
| SH3PXD2B | 613293 | chr5:171752162-171881552 | 8675 | SH3 AND PX DOMAINS-CONTAINING PROTEIN 2B |
| SHOX | 312865 | chrX:585054-620171 | 4734 | SHORT STATURE HOMEOBOX |
| SKI | 164780 | chr1:2160109-2241677 | 6057 | SKI PROTOONCOGENE |
| SLC17A5 | 604322 | chr6:74303077-74363762 | 3864 | SOLUTE CARRIER FAMILY 17 (ACIDIC SUGAR TRANSPORTER), MEMBER 5 |
| SLC26A2 | 606718 | chr5:149340275-149366988 | 8225 | SOLUTE CARRIER FAMILY 26 (SULFATE TRANSPORTER), MEMBER 2 |
| SLC29A3 | 612373 | chr10:73078985-73123172 | 2573 | SOLUTE CARRIER FAMILY 29 (NUCLEOSIDE TRANSPORTER), MEMBER 3 |
| SLC34A3 | 609826 | chr9:140125184-140131031 | 2953 | SOLUTE CARRIER FAMILY 34 (SODIUM/PHOSPHATE COTRANSPORTER), MEMBER 3 |
| SLC35D1 | 610804 | chr1:67464990-67520105 | 7060 | SOLUTE CARRIER FAMILY 35 (UDP-GLUCURONIC ACID/UDP-N-ACETYLGALACTOSAMINE DUAL TRANSPORTER), MEMBER D1 |
| SLC39A13 | 608735 | chr11:47428802-47438076 | 3258 | SOLUTE CARRIER FAMILY 39 (ZINC TRANSPORTER), MEMBER 13 |
| SLCO2A1 | 601460 | chr3:133651515-133748945 | 4923 | SOLUTE CARRIER ORGANIC ANION TRANSPORTER FAMILY, MEMBER 2A1 |
| SLCO5A1 | 613543 | chr8:70584543-70747324 | 4511 | SOLUTE CARRIER ORGANIC ANION TRANSPORTER FAMILY, MEMBER 5A1 |
| SMAD3 | 603109 | chr15:67358170-67487558 | 7223 | SMAD FAMILY MEMBER 3 |
| SMAD4 | 600993 | chr18:48556558-48611436 | 9371 | SMAD FAMILY MEMBER 4 |
| SMARCAL1 | 606622 | chr2:217277112-217347799 | 4268 | SWI/SNF-RELATED, MATRIX-ASSOCIATED, ACTIN-DEPENDENT REGULATOR OF CHROMATIN, SUBFAMILY A-LIKE PROTEIN 1 |
| SMC1A | 300040 | chrX:53401045-53449702 | 11230 | STRUCTURAL MAINTENANCE OF CHROMOSOMES 1A |
| SMC3 | 606062 | chr10:112327424-112364417 | 5562 | STRUCTURAL MAINTENANCE OF CHROMOSOMES 3 |
| SNRPB | 182282 | chr20:2442256-2451524 | 1503 | SMALL NUCLEAR RIBONUCLEOPROTEIN POLYPEPTIDES B AND B1 |
| SNX10 | 614780 | chr7:26331490-26413975 | 3612 | SORTING NEXIN 10 |
| SOST | 605740 | chr17:41831074-41836181 | 2400 | SCLEROSTIN |
| SOX9 | 608160 | chr17:70117136-70122585 | 4084 | SRY-BOX 9 |
| SP7 | 606633 | chr12:53720335-53738602 | 3517 | TRANSCRIPTION FACTOR Sp7 |
| SULF1 | 610012 | chr8:70378834-70573172 | 7602 | SULFATASE 1 |
| SUMF1 | 607939 | chr3:4402804-4508991 | 2611 | SULFATASE-MODIFYING FACTOR 1 |
| TBCE | 604934 | chr1:235530650-235612308 | 3123 | TUBULIN-SPECIFIC CHAPERONE E |
| TBX15 | 604127 | chr1:119425641-119532204 | 3895 | T-BOX TRANSCRIPTON FACTOR 15 |
| TBX3 | 601621 | chr12:115108034-115121994 | 5183 | T-BOX TRANSCRIPTION FACTOR 3 |
| TBX4 | 601719 | chr17:59533782-59561689 | 2870 | T-BOX TRANSCRIPTION FACTOR 4 |
| TBX5 | 601620 | chr12:114791710-114846272 | 4862 | T-BOX TRANSCRIPTION FACTOR 5 |
| TBX6 | 602427 | chr16:30097090-30103230 | 2252 | T-BOX TRANSCRIPTION FACTOR 6 |
| TBXAS1 | 274180 | chr7:139478022-139720150 | 3595 | THROMBOXANE A SYNTHASE 1 |
| TCF12 | 600480 | chr15:57210808-57580755 | 6063 | TRANSCRIPTION FACTOR 12 |
| TCIRG1 | 604592 | chr11:67806437-67818391 | 3991 | T CELL IMMUNE REGULATOR 1 |
| TCOF1 | 606847 | chr5:149737177-149779896 | 7394 | TCOF1 PROTEIN |
| TCTN3 | 613847 | chr10:97423128-97453925 | 3439 | TECTONIC FAMILY, MEMBER 3 |
| TGDS | 616146 | chr13:95226283-95248554 | 2596 | TDP-GLUCOSE 4,6-DEHYDRATASE |
| TGFB1 | 190180 | chr19:41836411-41859863 | 2933 | TRANSFORMING GROWTH FACTOR, BETA-1 |
| TGFB2 | 190220 | chr1:218518651-218617986 | 6354 | TRANSFORMING GROWTH FACTOR, BETA-2 |
| TGFBR1 | 190181 | chr9:101867346-101916498 | 6977 | TRANSFORMING GROWTH FACTOR-BETA RECEPTOR, TYPE I |
| TGFBR2 | 190182 | chr3:30647969-30735658 | 5103 | TRANSFORMING GROWTH FACTOR-BETA RECEPTOR, TYPE II |
| THPO | 600044 | chr3:184089698-184097501 | 2536 | THROMBOPOIETIN |
| TMEM216 | 613277 | chr11:61159807-61166360 | 1570 | TRANSMEMBRANE PROTEIN 216 |
| TMEM38B | 611236 | chr9:108456781-108538917 | 3843 | TRANSMEMBRANE PROTEIN 38B |
| TMEM67 | 609884 | chr8:94767047-94831485 | 6603 | TRANSMEMBRANE PROTEIN 67 |
| TNFRSF11A | 603499 | chr18:59992495-60054968 | 5093 | TUMOR NECROSIS FACTOR RECEPTOR SUPERFAMILY, MEMBER 11A |
| TNFRSF11B | 602643 | chr8:119935771-119964408 | 2596 | TUMOR NECROSIS FACTOR RECEPTOR SUPERFAMILY, MEMBER 11B |
| TNFSF11 | 602642 | chr13:43136847-43182174 | 3127 | TUMOR NECROSIS FACTOR LIGAND SUPERFAMILY, MEMBER 11 |
| TP63 | 603273 | chr3:189349191-189615093 | 7324 | TUMOR PROTEIN p63 |
| TRAPPC2 | 300202 | chrX:13730336-13752779 | 3162 | TRACKING PROTEIN PARTICLE COMPLEX, SUBUNIT 2 |
| TREM2 | 605086 | chr6:41126219-41130949 | 1305 | TRIGGERING RECEPTOR EXPRESSED ON MYELOID CELLS 2 |
| TRIP11 | 604505 | chr14:92434218-92506428 | 9138 | THYROID HORMONE RECEPTOR INTERACTOR 11 |
| TRPS1 | 604386 | chr8:116420699-116681280 | 10556 | ZINC FINGER TRANSCRIPTION FACTOR TRPS1 |
| TRPV4 | 605427 | chr12:110220867-110271237 | 4039 | TRANSIENT RECEPTOR POTENTIAL CATION CHANNEL, SUBFAMILY V, MEMBER 4 |
| TTC21B | 612014 | chr2:166729847-166810373 | 6927 | TETRATRICOPEPTIDE REPEAT DOMAIN-CONTAINING PROTEIN 21B |
| TWIST1 | 601622 | chr7:19155066-19157320 | 1766 | TWIST FAMILY bHLH TRANSCRIPTION FACTOR 1 |
| TYROBP | 604142 | chr19:36395278-36399236 | 841 | TYRO PROTEIN TYROSINE KINASE-BINDING PROTEIN |
| WDR19 | 608151 | chr4:39183999-39287455 | 6370 | WD REPEAT-CONTAINING PROTEIN 19 |
| WDR34 | 613363 | chr9:131395915-131419154 | 2268 | WD REPEAT-CONTAINING PROTEIN 34 |
| WDR35 | 613602 | chr2:20110004-20189909 | 8344 | WD REPEAT-CONTAINING PROTEIN 35 |
| WISP3 | 603400 | chr6:112375253-112390912 | 1776 | WNT1-INDUCIBLE SIGNALING PATHWAY PROTEIN 3 |
| WNT1 | 164820 | chr12:49372211-49376421 | 2484 | WINGLESS-TYPE MMTV INTEGRATION SITE FAMILY, MEMBER 1 |
| WNT10B | 601906 | chr12:49359098-49365666 | 2619 | WINGLESS-TYPE MMTV INTEGRATION SITE FAMILY, MEMBER 10B |
| WNT3 | 165330 | chr17:44839847-44896151 | 3605 | WINGLESS-TYPE MMTV INTEGRATION SITE FAMILY, MEMBER 3 |
| WNT5A | 164975 | chr3:55499718-55521695 | 6545 | WINGLESS-TYPE MMTV INTEGRATION SITE FAMILY, MEMBER 5A |
| WNT6 | 604663 | chr2:219724521-219738979 | 1900 | WINGLESS-TYPE MMTV INTEGRATION SITE FAMILY, MEMBER 6 |
| WNT7A | 601570 | chr3:13860057-13921643 | 1914 | WINGLESS-TYPE MMTV INTEGRATION SITE FAMILY, MEMBER 7A |
| XYLT1 | 608124 | chr16:17196156-17564763 | 9936 | XYLOSYLTRANSFERASE 1 |
| XYLT2 | 608125 | chr17:48423368-48438571 | 4151 | XYLOSYLTRANSFERASE 2 |
| ZMPSTE24 | 606480 | chr1:40723697-40759881 | 3665 | ZINC METALLOPROTEINASE STE24 |
