# Supplementary material for: Genetics Evaluation of Targeted Exome Sequencing in 223 Chinese Probands With Genetic Skeletal Dysplasias
Source: Front Cell Dev Biol. 2021 Sep 7;9:715042. doi: 10.3389/fcell.2021.715042 (PMC8452955; doi:10.3389/fcell.2021.715042)
Supplement: Supplementary Table 3 — Quality metrics of targeted panel sequencing. [file Table_3.DOCX]

| Supplementary Table S3. Quality metrics of targeted panel sequencing | |
| --- | --- |
| Sequencing Metrics | Mean ± SD |
| Total reads | 6933449.366±1820819.661 |
| Mapped reads | 3466724.683±910409.8307 |
| Mean bait coverage | 335.9708954±82.80363804 |
| Target bases covered 10X | 93.97% |
| Target bases covered 30X | 89.73% |
| Target bases covered 50X | 86.22% |
| reads Q30 | 91.21% |
